# A nomogram prediction model for embryo implantation outcomes based on the cervical microbiota of the infertile patients during IVF-FET

**DOI:** 10.1128/spectrum.01462-24

**Published:** 2025-03-07

**Authors:** Yanan Wu, Lingyun Shi, Zili Jin, Wenjun Chen, Fuxin Wang, Huihua Wu, Hong Li, Ce Zhang, Rui Zhu

**Affiliations:** 1 Center for Human Reproduction and Genetics, Affiliated Suzhou Hospital of Nanjing Medical University, Suzhou Municipal Hospital, Gusu School, Nanjing Medical University, Suzhou, China; 2 State Key Laboratory of Reproductive Medicine and Offspring Health, Nanjing Medical University 12461 , Nanjing, China; University of Rome, Rome, Italy

**Keywords:** female infertility, cervical microbiota, embryo implantation, *Halomonas*, *in vitro* fertilization-frozen embryo transfer (IVF-FET)

## Abstract

The microbiota of the female genital tract is crucial for reproductive health. This study aims to investigate the impact of the lower genital tract microbiota on *in vitro* fertilization and frozen embryo transfer (IVF-FET) outcomes. This study included 131 women aged 20–35 years who underwent their first or second IVF-FET cycle with no obvious unfavorable factors for implantation. Cervical microbiota samples were collected on the embryo transfer day and analyzed using 16S rDNA full-length sequencing. Clinical outcomes were followed up for analysis. Clinical pregnancy (CP) was achieved in 84 patients, and 47 patients experienced non-pregnancy (NP). The cervical microbiota diversity between NP and CP groups showed no significant differences, but some genera such as *Halomonas* (*P* = 0.018), *Klebsiella* (*P* = 0.039), *Atopobium* (*P* = 0.016), and *Ligilactobacillus* (*P* = 0.021) were obviously different between the two groups. Notably, there was no significant difference in the abundance of *Lactobacillus* between the two groups. A nomogram prediction model was developed using the random forest algorithm and logistic regression, including the classification of *Halomonas*, *Atopobium*, and *Veillonella*, as well as the relative abundance of *Lactobacillus*, to identify high-risk patients with embryo implantation failure. Both internal (area under the curve [AUC] = 0.718, 95% confidence interval [CI]: 0.628–0.807, *P* < 0.001) and external validation (AUC = 0.654, 95% CI: 0.553–0.755, *P* = 0.037) of the model performed well. In conclusion, this study established a correlation between cervical microbiota and embryo implantation failure in infertile women undergoing IVF-FET and developed a prediction model that could help in early identification of patients at high risk of implantation failure.

**IMPORTANCE:**

This study investigated the potential role of abnormal cervical microbiota in the pathology of implantation failure after *in vitro* fertilization and frozen embryo transfer (IVF-FET) treatment. Despite nearly half a century of advancements in assisted reproductive technology (ART), the implantation rate of high-quality embryos still hovered around 50%. Moreover, unexplained recurrent implantation failure (RIF) remains a significant challenge in ART. To our knowledge, we first discovered a prediction model for embryo implantation failure, identifying *Halomonas* and *Veillonella* as significantly adverse factors for embryo implantation. Despite some limitations, the internal and external validation of the model could bode well for its clinical application prospect. The insights gained from this study pave the way for intervention in the genital tract microbiota prior to IVF-FET, particularly in patients with RIF and RSA.

## INTRODUCTION

Infertility is defined as the inability of a couple of childbearing age to become pregnant after having regular unprotected intercourse for at least 1 year (1). The incidence of infertility among reproductive-aged couples in China has reached 17.6% (2), making it a global public health issue alongside cancer and cardiovascular disease. *In vitro* fertilization and embryo transfer (IVF-ET) is widely regarded as a valid method for treating infertility, with the cumulative pregnancy rates after three cycles still hovering around 50% even after nearly half a century of advancements in assisted reproductive technology (ART) (3). However, recurrent implantation failure (RIF) and recurrent spontaneous abortion (RSA) remain significant challenges in ART as their causes are often unknown and complex. Approximately 10% of women undergoing three cycles of embryo transfer experience RIF, and 12%–15% of women experience miscarriage per transfer cycle, creating a distressing and frustrating situation (4). Among various factors, the reproductive tract microenvironment, particularly the genital tract microbiome, plays a vital role in establishing and maintaining pregnancy (5, 6).

The human body harbors a vast number of microbes (7). Approximately 9% of the microbiome exists in the female reproductive tract and has a significant impact on female reproductive health (8, 9). A healthy vaginal microbiome is typically defined as a *Lactobacillus*-dominant population, while a non-*Lactobacillus*-dominant vaginal microbiota is generally associated with a high Nugent score and bacterial vaginosis (BV) (10, 11). In healthy women, the ratio of *Lactobacillus* located in the vaginal microbiota is usually greater than 90% and can be classified into four types of *Lactobacillus* spp. (*Lactobacillus crispatus*, *Lactobacillus jensenii*, *Lactobacillus iners*, and *Lactobacillus gasseri*). Among these, *L. crispatus* is considered a vaginal genus with healthy outcomes, while *Lactobacillus iners* is associated with poor outcomes, such as BV, HPV infection, embryo implantation failure, and other disorders (10, 12–16). More precise research on the microbiota located in the cervix or uterus has drawn public attention. *Lactobacillus*, which is present in the upper reproductive tract, has been found to promote a healthy reproductive tract environment. Conversely, uterine dysbiosis is indicative of a series of disorders, such as chronic endometritis (CE) and RSA (17–20).

The balanced symbiotic barrier formed by the cervico-vaginal epithelium and *Lactobacillus* helps protect against the invasion of pathogens (21, 22). When this balance is disrupted, it can lead to poor reproductive outcomes, such as sexually transmitted diseases, pelvic inflammatory diseases, infertility, and miscarriage (23–28). Furthermore, it has been found to be related to the establishment of pregnancy after IVF-ET. According to Haahr’s research, a negative clinical pregnancy was associated with an aberrant vaginal microbiota due to BV (29). Microorganisms such as *Bifidobacterium*, *Prevotella*, *Lactobacillus iners*, and *Streptococcus* were verified to lead to pregnancy failure (30–32). Compared to individuals with non-*Lactobacillus*-dominant microbiota (NLDM) (< 90% *Lactobacillus*), the clinical pregnancy rate of individuals with *Lactobacillus*-dominant microbiota (LDM) (≥ 90% *Lactobacillus*) is significantly higher (19, 33). Moreover, *Lactobacillus* may also serve as a tool for predicting favorable outcomes of IVF-ET (20, 32, 34).

However, other researchers have reported controversial findings. Ruiying Wang *et al*. found no obvious difference in the abundance of *Lactobacillus* in the cervix and uterus between pregnant and nonpregnant individuals who underwent IVF-ET treatment (32). According to LelaK. Keburiya’s study, there was also no striking difference in the abundance of *Lactobacillus* in the uterine microbiota between patients who received embryo transfer for the first time and those who experienced RIF. Additionally, although a significant difference was identified in the prevalence of strict anaerobes and *Gardnerella* in Keburiya’s study, it had no deleterious effect on pregnancy rates (35). Therefore, more rigorously designed research studies are necessary to determine whether the microbiota of the female genital tract influences the outcomes of IVF-ET.

Advancing our understanding of microbial etiologies in the lower genital tract of females with adverse post-IVF-FET outcomes, this study aimed to explore the role of cervical microbiota anomalies in embryo implantation failure and to develop a predictive model for embryo implantation failure based on cervical microbiota profiles.

## RESULTS

### Cervical microbiota diversity and composition analysis

**Fig 1:**
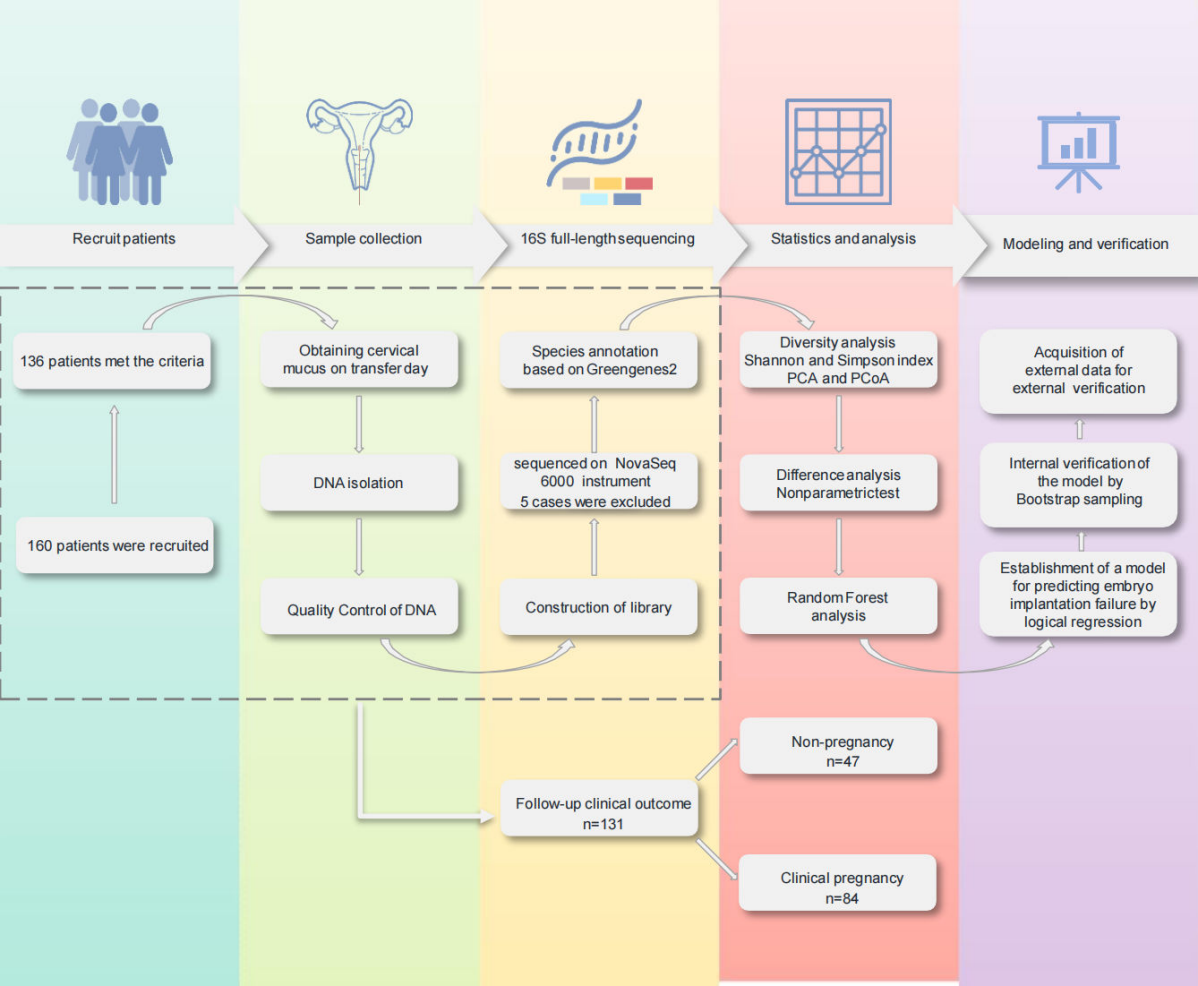
Schematic illustration of the study design.

A flow diagram illustrating the study design is provided in Fig. 1. Among the 131 patients enrolled, 47 experienced embryo implantation failure, indicating non-pregnancy (NP), and 84 cases achieved clinical pregnancy (CP). The clinical characteristics of the enrolled subjects are detailed in Table 1.

**TABLE 1:** Baseline and transplantation characteristics of enrolled patients^*d*^

	NP group	CP group		
Characteristic	(*n* = 47)	(*n* = 84)	t/X^2^ value	*P* value
Age at last birthday—yr (mean ± SD)	29.45 ± 3.236	30.04 ± 3.232	1.000	0.319^*a*^
Body mass index—kg/m2 (mean ± SD)	23.06 ± 3.778	22.50 ± 2.875	0.895	0.374^*a*^
Infertility years—yr (mean ± SD)	4.38 ± 2.882	4.29 ± 2.366	0.180	0.857^*a*^
Basic estradiol—pg/mL (mean ± SD)	35.32 ± 14.706	35.88 ± 20.542	0.162	0.872^*a*^
Basal progesterone—ng/mL (mean ± SD)	0.61 ± 0.386	0.59 ± 0.359	0.256	0.798^*a*^
Basal follicle-stimulating hormone—mIU/mL (mean ± SD)	7.30 ± 2.078	7.46 ± 2.075	0.431	0.667^*a*^
Anti-Mullerian hormone—ng/mL (mean ± SD)	6.32 ± 4.394	6.33 ± 4.654	0.017	0.986^*a*^
Menstrual cycle—no. (%)				
Regular	29 (61.70)	45 (53.57)	0.811	0.368^*b*^
Irregular	18 (38.30)	39 (46.43)		
Previous pregnancy history—no. (%)				
No	24 (51.06)	51 (60.71)	1.147	0.284^*b*^
Yes	23 (48.94)	33 (39.29)		
Embryonic stage—no. (%)				
Cleavage stage embryo	10 (21.28)	30 (35.71)	2.962	0.085^*b*^
Blastocyst	37 (78.72)	54 (64.29)		
Endometrial thickness on transplantation day—mm (mean ± SD)	8.69 ± 1.038	8.89 ± 1.429	0.866	0.388^*a*^
Protocols of frozen-thawed cycles—no. (%)				
Downregulate hormone replacement cycle	10 (21.28)	25 (29.76)	NA^*e*^	0.193^*c*^
Hormone replacement cycle	27 (57.44)	49 (58.33)		
Natural cycle	10 (21.28)	8 (9.53)		
Ovulation induction cycle	0 (0.00)	2 (2.38)		
Estrogen on day 14 after transplantation—pg/mL (mean ± SD)	304.48 ± 360.943	275.04 ± 316.543	0.478	0.633^*a*^
Progesterone on day 14 after transplantation—ng/ml (mean ± SD)	11.05 ± 8.742	9.20 ± 6.398	1.369	0.173^*a*^

^*a*^ By *t*-test.

^*b*^ By chi-square test.

^*c*^ By Fisher exact test.

^*d*^ NP, non-pregnancy; CP, clinical pregnancy; values are given as mean ± SD, number (%).

^*e*^ NA, not applicable.

**Fig 2:**
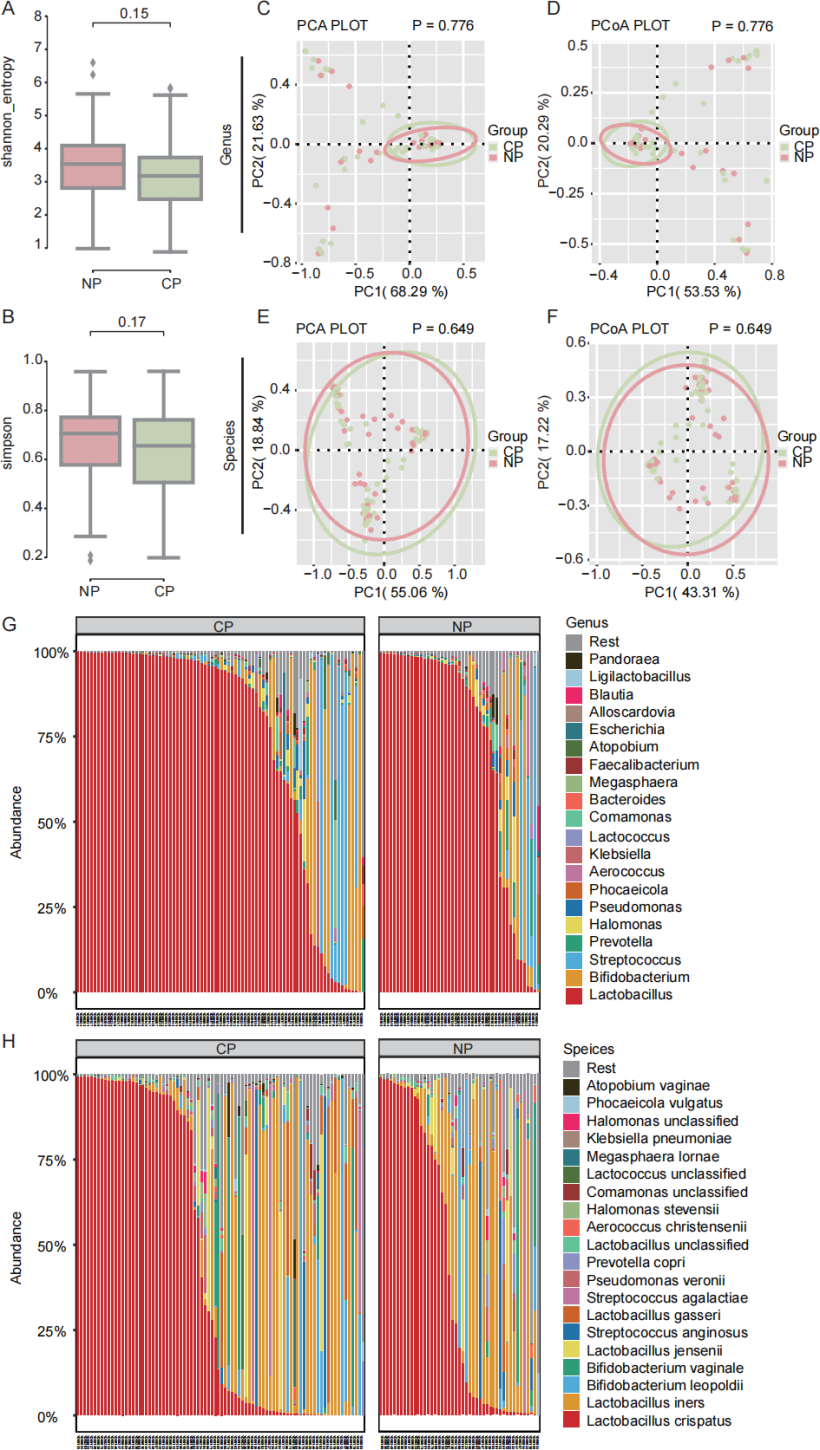
Diversity and composition of the cervical microbiota. (**A, B**) Alpha-diversity of the cervical microbiota in the two groups was calculated and is shown by the Shannon and Simpson indexes. (**C, E**) Principal component analysis (PCA) illustrated the distribution of the cervical microbiota at the genus and species levels. (**D, F**) Principal coordinate analysis (PCoA) represented the distribution of the cervical microbiota at the genus and species levels. (**G**) Genus-level composition, highlighting the 20 most abundant genera. (**H**) Species-level composition, highlighting the 20 most abundant species. NP, non-pregnancy; CP, clinical pregnancy.

A total of 4,299 operational taxonomic units (OTUs) were identified in the cervical microbiota. The alpha diversity of the microbiota was calculated by the Shannon index (*P* value = 0.15) and Simpson index (*P* value = 0.17) (Fig. 2A and B). No significant differences in the alpha diversity were observed between the NP and CP groups. Principal component analysis (PCA) and principal coordinate analysis (PCoA) performed at the phylum level showed no significant differences between the two groups (Fig. S1). Further PCA (Fig. 2C and E) and PCoA (Fig. 2D and F) were applied to illustrate the distribution of the microbial community in the samples. The beta diversity analysis at both the genus and species levels revealed no significant differences between the NP and CP groups. A high level of overlap between the two groups of samples was observed.

*Firmicutes* was the dominant bacteria of the two groups of cervical microbiota at the phylum level (Fig. S2A). No statistically significant differences were shown in *Firmicutes* (*P* = 0.291), with the mean relative abundance of *Firmicutes* in the NP and CP groups being 83.377% and 84.020%, respectively. *Firmicutes* was followed by *Actinobacteriota*, *Proteobacteria*, *Bacteroidota*, and other taxa. However, only *Fusobacteriota* showed significant differences between the NP group and the CP group at the phylum level (0.204% vs. 0.024%, *P* = 0.008). The composition of other levels of bacteria is shown in Fig. S2. Comparative results of the relative abundance of bacteria at other levels are also shown in Table S1.

The composition of the two groups is similar at the genus and species levels. Figure 2G displays the top 20 most abundant genera. Among them, *Halomonas*, *Klebsiella*, *Atopobium*, and *Ligilactobacillus* were obviously different between the two groups. The relative abundances of *Lactobacillus*, *Bifidobacterium*, and *Streptococcus* exceeded 1% in both groups, while those of *Prevotella*, *Halomonas*, and *Aerococcus* were higher than 1% only in the NP group. A total of 21 genera were found to have significant differences between the two groups (Table S1). At the species level, species with the top 20 abundances are shown in Fig. 2H. Among them, *Streptococcus agalactiae*, *Prevotella copri*, *Halomonas stevensii*, *Megasphaera lornae,* and *Atopobium vaginae* had obvious significant differences between the two groups. The relative abundances of *L. crispatus, Lactobacillus iners, Bifidobacterium leopoldii, Bifidobacterium vaginale, Lactobacillus jensenii, Streptococcus anginosus,* and *Streptococcus agalactiae* were higher than 1% in both groups, while those of *Prevotella copri* and *Aerococcus christensenii* were more than 1% only in the NP group. A total of 44 species differed significantly between the two groups (Table S1).

### Random forest analysis

**Fig 3:**
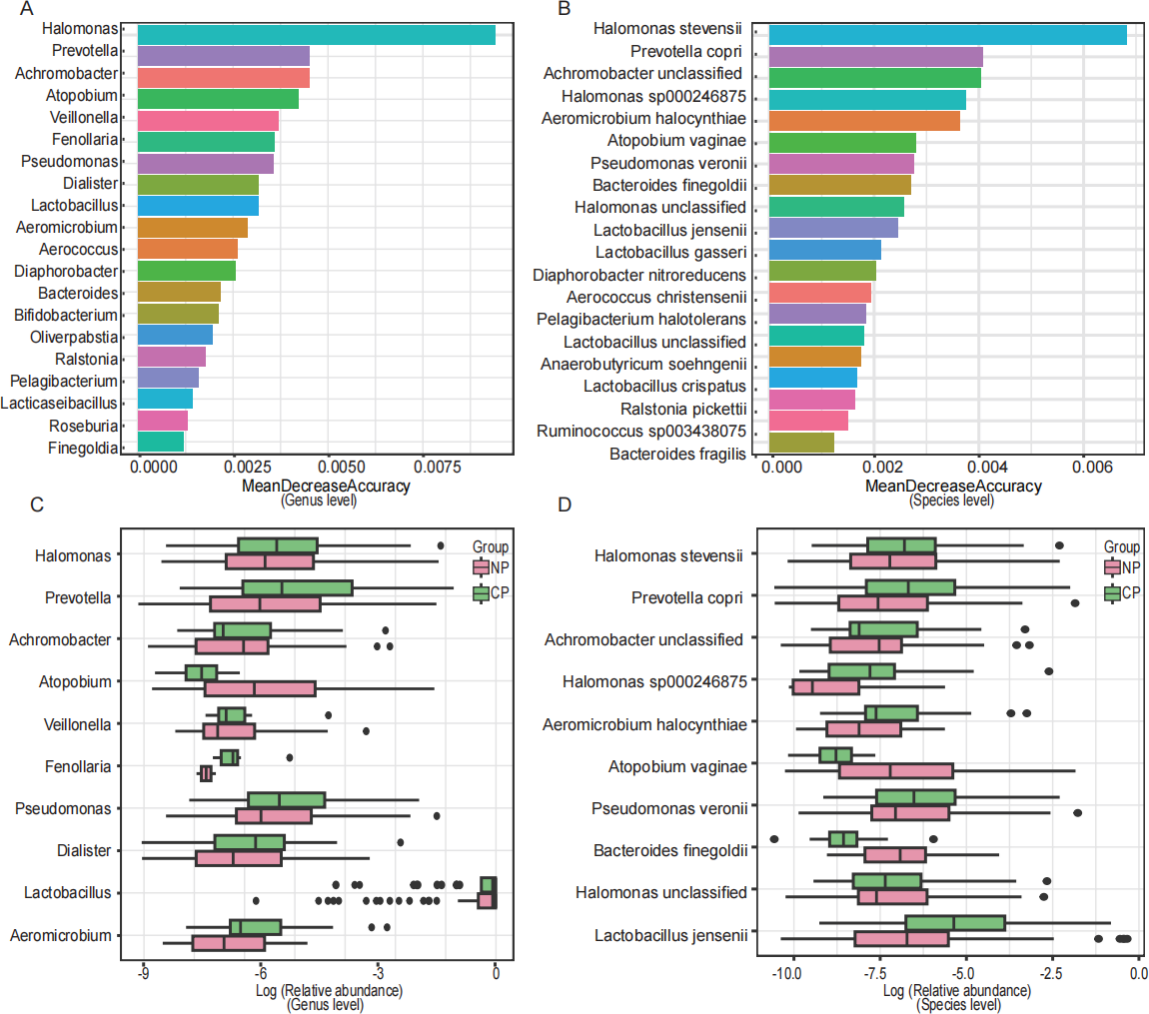
Random forest analysis. (**A, B**) Random forest analysis was conducted to map the importance of genera or species, with the horizontal axis representing the importance measure and the vertical axis listing the genera or species ranked by their importance. (**C, D**) Boxplots are presented for the genera or species that ranked among the top ten based on the random forest analysis. NP, non-pregnancy; CP, clinical pregnancy.

Further, the random forest algorithm identified key microbial biomarkers influencing embryo implantation. The model consists of 1,000 decision trees, each built on a different random subset of the training data. Feature importance was assessed by mean decrease accuracy (MDA). The results showed that *Halomonas* was the most significant contributor to the model at the genus level, with the highest MDA, indicating that it played a key role in the classification process (Fig. 3A). *Prevotella*, *Achromobacter*, *Atopobium*, *Veillonella*, *Fenollaria*, *Pseudomonas*, *Dialister*, *Lactobacillus,* and *Aeromicrobium* also showed higher MDA values, indicating that they also contributed to the model (Fig. 3A). The box plot showed the top 10 genera with the highest MDA values (Fig. 3C). At the species level, the highest contribution to the model was from *Halomonas stevensii*, followed by *Prevotella copri*, *Achromobacter unclassified*, *Halomonas sp000246875*, *Aeromicrobium halocynthiae*, *Atopobium vaginae*, *Pseudomonas veronii*, etc. (Fig. 3B). Figure 3D shows the box plots of the top 10 species with MDA scores.

### Establishment and validation of a prediction model for embryo implantation failure

**Fig 4:**
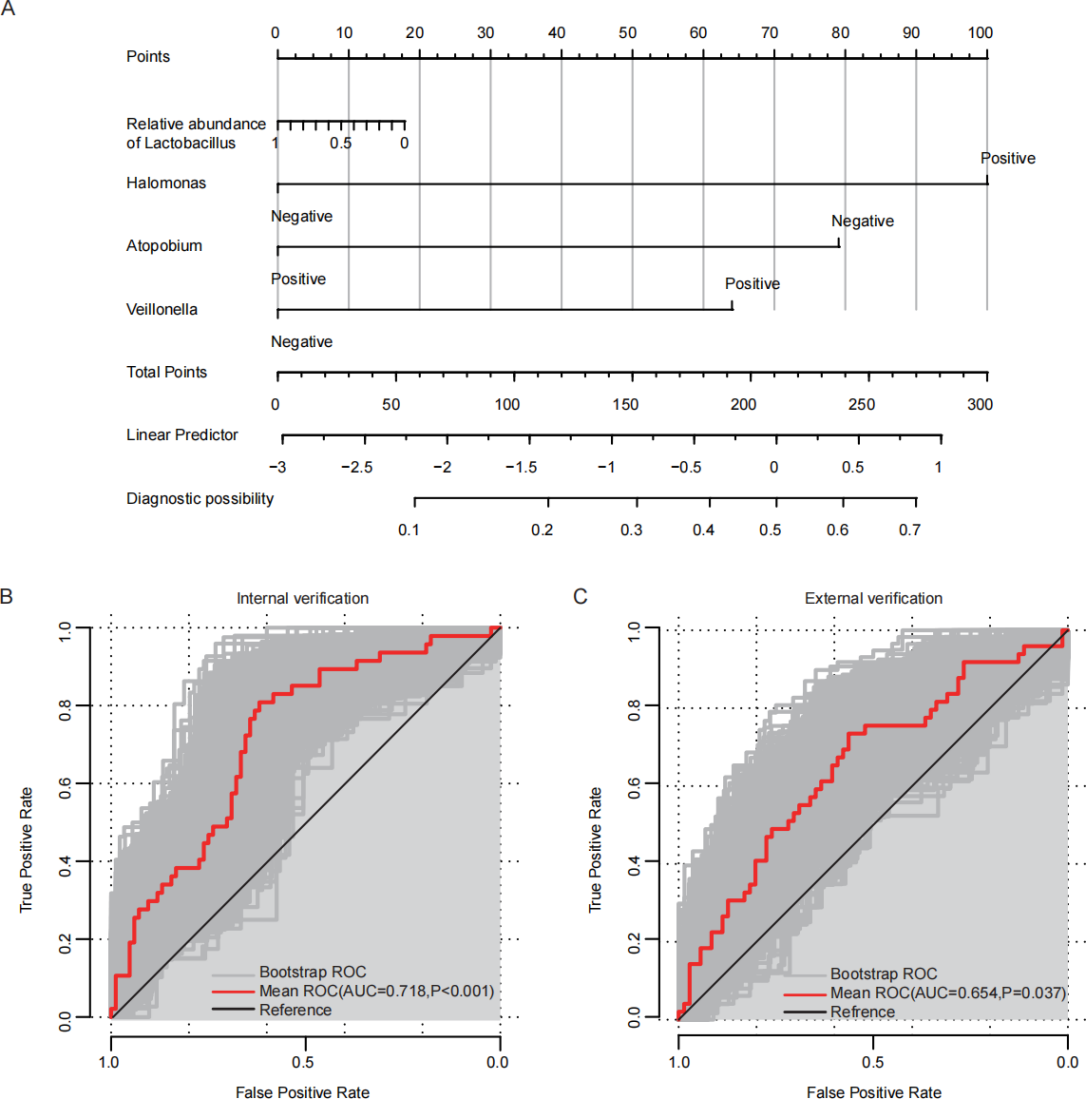
Prediction nomogram for embryo implantation failure and ROC curves for internal and external model validation using bootstrap sampling. (**A**) Nomogram of the embryo implantation failure prediction model. (**B**) ROC curve of internal verification through bootstrap sampling. (**C**) ROC curve of external verification through bootstrap sampling.

The top 10 genera, based on MDA scores from the random forest analysis, were incorporated as categorical predictors (positive vs negative) in the multivariate binary logistic regression model. The backward LR method was used for stepwise regression, and the relative abundance of *Lactobacillus* was a must for inclusion in the model. The final prediction model consisted of the classifications of *Halomonas*, *Atopobium*, and *Veillonella*, as well as the relative abundance of *Lactobacillus* (𝐿𝑜𝑔𝑖𝑡(𝑝) = −1.673 + (−0.257 × 𝑟𝑒𝑙𝑎𝑡𝑖𝑣𝑒 𝑎𝑏𝑢𝑛𝑑𝑎𝑛𝑐𝑒 𝑜𝑓 𝐿𝑎𝑐𝑡𝑜𝑏𝑎𝑐𝑖𝑙𝑙𝑢𝑠) + 1.436 × classification of 𝐻𝑎𝑙𝑜𝑚𝑜𝑛𝑎𝑠 + (−1.136 × classification of 𝐴𝑡𝑜𝑝𝑜𝑏𝑖𝑢𝑚) + 0.92 × classification of 𝑉𝑒𝑖𝑙𝑙𝑜𝑛𝑒𝑙𝑙𝑎)) (Fig. 4A). In order to assess the robustness and generalization ability of the model, the bootstrap method was used for internal validation. The average ROC curve of internal validation is displayed in Fig. 4B (AUC = 0.718, 95% CI: 0.628–0.807, *P* < 0.001).

In order to evaluate the generalization ability of the predictive model, further external validation was carried out. An independent data set from the project PRJNA903403 (20), containing 16S full-length sequencing data from the cervical microbiota of 120 patients undergoing IVF treatment, was used. A comparison of internal and external data is shown in Table 2. The bootstrap method was also used to verify the external data, and the results indicated that the mean ROC curve of the external data had an AUC greater than 0.6 (AUC = 0.654, 95% CI: 0.553–0.755, *P* = 0.037) (Fig. 4C). Slight differences existed in the characteristics of the subjects in the external data and the subjects in this study. Despite these discrepancies, the model demonstrated robust predictive accuracy in external validation, underscoring its clinical application.

**TABLE 2:** Comparison of internal and external data groups^*d*^

	Internal	External
Item	*n* = 131	*n* = 120
Location	Jiangsu Province, China	Liaoning Province, China
Age at last birthday	20–35 yr	20–40 yr
Body mass index	Less than or equal to 30 kg/m^2^	Unrestricted
Transfer cycle	Frozen embryo transfer	Frozen embryo transfer
Luteal support	Unrestricted	Hormone replacement therapy
Quality and quantity of embryos	High-quality cleavage stage or blastocyst transferred, and the number of embryos is specified.	High-quality cleavage stage or blastocyst transferred
Endometrial thickness on the transfer day	Not less than 7 mm	Greater than 8 mm
History of antibiotic use	Not in the past 2 months	Not in the past 1 month
Exclusion criteria		
Severe hydrosalpinx	Y	N
Stage III–IV endometriosis or severe adenomyosis	Y	Y^*a*^
Moderate-to-severe intrauterine adhesions without restoration by hysteroscopy	Y	Y^*a*^
Primary uterine malformation	Y	Y
RSA or RIF	Y	Y^*b*^
Autoimmune disease	Y	N
Diabetes	Y	N
Thrombotic diseases	Y	N
Anti-Müllerian hormone < 1.1 ng/mL	Y	N
Smokers or alcoholics	N^*c*^	Y
Participation in any experimental drug study within 60 days	N	Y

^*a*^ No severity specified.

^*b*^ Three embryo implantation failures were taken as the RIF standard, and RSA is not included.

^*c*^ No specific requirements are made, but smokers or alcoholics are not included in the internal data.

^*d*^ Y, yes; N, no.

## DISCUSSION

In this study, the association between the cervical microbiome and embryo implantation fate was investigated. Additionally, we developed a clinical prediction model based on the cervical microbiota for the early identification of high-risk patients with embryo implantation failure. According to our findings, the composition of the cervical microbiota was associated with embryo implantation. Although we found no significant difference in the diversity of the cervical microbiota between the NP and CP groups, specific bacteria, including *Halomonas*, *Klebsiella*, *Atopobium*, and *Ligilactobacillus*, showed significant differences between the two groups. The genera that significantly contributed to differentiating NP and CP in our model were identified using a random forest algorithm. The prediction model included classifications of *Halomonas*, *Atopobium*, and *Veillonella*, along with the relative abundance of *Lactobacillus*. Both the internal and external validation of the model showed an AUC greater than 0.6, indicating its potential value in clinical applications.

Successful implantation and healthy growth of the embryo are facilitated by potential embryos, a receptive uterus, and the harmonious interaction between them. As is well known, the quality and quantity of embryos are of great importance to the first step of pregnancy. Previous studies showed that the number of high-quality embryos is an important predictor of CP (36). In addition, embryonic chromosomal abnormalities are important factors that prevent early pregnancy from being established and maintained. A chromosomal error originating from an oocyte can lead to the creation of aneuploid embryos, which are age-related to the female, especially with advanced age over 38 years (37). In this study, all embryos were of good quality, the stage and number of embryos transferred were equivalent, and the age of women was less than 35 years, eliminating interfering factors as much as possible. Another important factor affecting embryo implantation is the receptive endometrium, and the substantial impacts exerted by the female anatomic structure of the uterus and hormone fluctuations on the endometrium play critical roles in the survival of pregnancy (38, 39). Besides, a normal pregnancy is a successful homogeneous hemigenetic transplant, and maternal–fetal immune tolerance allows the embryo to successfully implant and develop until delivery. The substantial changes to the microbial communities inhabiting the mother’s genital tract participate in inducing maternal–fetal immune tolerance. *Gardnerella vaginalis* activates the production of more NF-κB and IL-8 in the cervico-vaginal epithelium, whereas *L. crispatus* does not (40). Patients with embryonic miscarriage have a lower abundance of *Lactobacillus* in the vagina and higher levels of IL-2 and IL-2/IL-10 in the vaginal lavage fluid (41). This evidence suggests that microorganisms in the reproductive tract may affect the local immune microenvironment by modulating the host immune response, resulting in an adverse influence on the endometrial receptivity.

The interplay between pregnancy and the microbiome has profound implications for maternal and infant wellbeing. *Lactobacillus* emerges as the sustainer of a balanced reproductive tract microbiota in healthy women. It can prevent genital tract infections in a variety of ways. Lactic acid has been shown to have antibacterial properties, and *Lactobacillus* is capable of producing large amounts of it (42). Competing with other bacteria, *Lactobacillus* can also prevent infection by directly adhering to host cells and regulating the secretion of cytokines by host cells (43, 44). In general, a disordered microbiota of the reproductive tract refers to the absence or decrease of *Lactobacillus*, which can lead to poor reproductive outcomes such as BV, sexually transmitted diseases, pelvic inflammatory diseases, infertility, and miscarriage (23–28). For infertile individuals, some previous studies have found that a *Lactobacillus*-dominant reproductive tract microbiota is associated with successful pregnancy after IVF treatment. Hong Zeng *et al.* (45) showed that the clinical pregnancy rate was greatly reduced in the non-*Lactobacillus*-dominant vaginal microbiota group (40.98% vs 50.82%, *P* < 0.01), consistent with the findings in endometrial microbiota from extensive studies (31, 46, 47). According to the studies of Sofia Väinämö (48) and Wenzheng Guan (20), *L. crispatus* may be the main contributor to clinical pregnancy and live birth.

In this research, the relative abundance of *Lactobacillus* did not exhibit a significant variation between the NP and CP groups. This finding is consistent with the studies conducted by Ruiying Wang (32) and Lela K. Keburiya (35). Wang reported that the microbiota of both the vagina and cervix did not show significant differences between the pregnant and nonpregnant groups who underwent fresh cleavage-stage embryo transfer (ET) in their first IVF/ICSI cycle. In Keburiya’s research, the relative abundance of *Lactobacillus* in the cervix did not vary significantly between the pregnant and nonpregnant groups, which included participants who received their first frozen embryo transfer (FET) or suffered from RIF. It remains controversial whether *Lactobacillus* has a significant effect on pregnancy outcomes after fresh or frozen ET, and further research studies are required to validate this conclusion. Here, we included the relative abundance of *Lactobacillus* in the model despite no prominent difference in its relative abundance between the NP and CP groups. The results showed that the model worked well on both the internal and the external data. In contrast, if the relative abundance of *Lactobacillus* was deleted from the model, less favorable performance was manifested on the external data. Based on these findings, *Lactobacillus* contributes to the establishment of pregnancy after IVF treatment, although it is not the primary factor. The presence of other pathogenic bacteria should also be taken into account.

In the established prediction models, *Halomonas* contributed the most and was significantly positively correlated with embryo implantation failure. *Halomonas,* a class of halophilic bacteria found in high-salt environments, is primarily used in biotechnology and environmental remediation due to its biological properties (49, 50). Originally considered an environmental microbe, *Halomonas* has been found in the human microbiota with the advancement of microbiota studies and has been linked to diseases such as *Helicobacter pylori* infection, breast cancer, and psychiatric disorders (51–53). *Halomonas* has also been detected in the female genital tract and is associated with early pregnancy miscarriage (54). There are few studies on *Halomonas* and female reproductive health, which may be partly due to the imprecision of the microbiota identification methods used in previous studies. To our knowledge, this study is the first to point out that *Halomonas* may be involved in embryo implantation failure, and further large-scale research is needed to validate this finding. *Veillonella*, a common bacterium found in the human oral, intestinal, and respiratory tracts, is associated with various diseases, including periodontal disease, chronic obstructive pulmonary disease (COPD), and inflammatory bowel disease (55–57). *Veillonella* in the female reproductive tract has been concerned with diseases such as cervical intraepithelial neoplasia, endometriosis, BV, and postpartum fever (58–61). Min Fu *et al*. found *Veillonella* as a potential biomarker for RIF development through a study of the vaginal microbiota of patients in the control and RIF groups (33). Here, we likewise reported the relationship between *Veillonella* and embryo implantation failure. *Atopobium* is a common genus in the female genital tract, previously considered the pathogenic bacteria of BV and preterm birth. Since it is also present in the normal microbiota of the female genital tract (62, 63), the presence of *Atopobium* does not mean that it is related to embryo implantation failure, and in most studies, only an abnormal increase in the abundance of *Atopobium* increased the high risk of embryo implantation failure. In our prediction model, the presence of *Atopobium* was found to be a protective factor for successful embryo implantation. Notably, the average relative abundance of *Atopobium* among the enrolled patients was less than 1%. Thus, our findings suggest that *Atopobium* emerges as a beneficial factor for embryo implantation. However, the potential negative impact of an abnormal increase in its abundance on embryo implantation requires further investigation.

Despite these strengths, some limitations of the study should be acknowledged. First, the sample size was relatively small, which may limit the generalizability of the findings. Second, due to the single-center design, selection bias cannot be completely eliminated. Third, although full-length 16S-rDNA provided more comprehensive information than traditional 16S rRNA gene sequencing, it still has limited resolution in identifying bacteria at the species or strain level. Fourth, we only collected cervical samples on the day of embryo transfer and could not assess the temporal changes of the microbiota during the course of pregnancy. Fifth, we did not account for some potentially contradictory factors such as diet and lifestyle, which may influence the cervical–vaginal microenvironment. In the future, large-scale, multicenter prospective studies are needed to validate these findings. More advanced sequencing technologies such as whole-genome shotgun metagenetics may be applied to gain deeper insights into the reproductive microbiota related to pregnancy outcomes. In addition to clinical factors, more comprehensive information on demography, socioeconomic status, and lifestyle should be collected to better control for confounding effects.

In conclusion, our study provides preliminary evidence of the correlation between cervical microbiota and embryo implantation failure. A prediction model is developed, which consists of the classification of *Halomonas*, *Atopobium,* and *Veillonella* and the relative abundance of *Lactobacillus*, with generalizability for external verification. These findings strengthen our understanding of the role of reproductive tract microbes in infertile females attending IVF-FET. The insights gleaned from this study may shed light on the evaluation and early intervention for patients with high risk of embryo transfer failure during IVF-FET.

## MATERIALS AND METHODS

### Study population

A total of 160 patients were recruited between November 2021 and August 2022. These patients planned to undergo *in vitro* fertilization (IVF) or intracytoplasmic sperm injection (ICSI), followed by frozen embryo transfer (FET) treatment at the reproductive center, which is located at the affiliated Suzhou hospital of Nanjing Medical University. Twenty-four patients were excluded because they did not meet the inclusion criteria.

Inclusion criteria:

1. Age between 20 and 35 years;
2. First or second FET cycle;
3. Quality and quantity of embryos: one high-quality blastocyst (≥4 BB), two D3 cleavage stage embryos (6–10 cells, with fragments ≤ 10% and symmetric blastomeres), or one D3 cleavage stage embryo with good quality, as determined by time-lapse observation;
4. Endometrial thickness not less than 7 mm on the transfer day;
5. No history of acute reproductive tract infection and no use of antibiotic use in the past 2 months.

Exclusion criteria:

1. Severe hydrosalpinx;
2. Stage III–IV endometriosis or severe adenomyosis;
3. Moderate-to-severe intrauterine adhesions without restoration by hysteroscopy;
4. Primary uterine malformation;
5. Body mass index (BMI) >30 kg/m2;
6. RSA or RIF;
7. Autoimmune disease;
8. Diabetes;
9. Thrombotic diseases;
10. Anti-Müllerian hormone <1.1 ng/mL.

### Sample collection and DNA isolation

Cervical mucus was collected on the day of FET by gently rotating a sterile cotton swab in the cervical canal after wiping away any surface secretions. To prevent contamination, the sampling swab should not come into contact with any other parts during this process. The samples were immediately immersed in liquid nitrogen and promptly transported to the laboratory for bacterial DNA extraction (Qiagen, Hilden, Germany). To trace the cervical microbiota, qPCR and 16S rDNA full-length sequencing were performed using this genomic DNA.

### 16S rDNA Full-length Sequencing

In this study, 16S full-length assembly sequencing technology (16S-FLAST) was adopted. The fundamental principles and schemes of FLAST are consistent with those depicted in earlier reports on the construction of a full-length single-molecular library of 16S rDNA genes (64). All samples are sequenced on the NovaSeq 6000 instrument (Illumina). About 496.9 Gb of data (1.7 billion reads) were obtained for 136 vaginal samples. According to the unique tag encoded in both flanks, we obtained 2,014,560 unique 16S rRNA gene sequences (>1,200 bp), among which, five samples were excluded because the sequence number was lower than 1,000 base pairs (bp). The full-length 16S rDNA gene sequences were obtained from the sequenced data by FLAST, and then these sequences were classified into operational taxonomic units (OTUs) using vSearch in QIIME2 with a similarity of 99% (65, 66). We used Mothur v1.42.0 (67) to annotate these OTUs in accordance with the reference database Silva_132_SSURef_Nr99 (68).

### Reproductive outcome measurements

Non-pregnancy was defined as a human chorionic gonadotropin (hCG) level <25 mIU/mL 14 days after FET. Conversely, if the hCG value exceeded 25 mIU/mL, biochemical pregnancy was generally accepted. Clinical pregnancy was determined by the presence of a gestational sac revealed by ultrasonography approximately 30 days after FET.

### Statistical analysis

Categorical variables were expressed as numbers (relative frequency %), while continuous variables were shown as mean ± standard deviation (SD). Student’s *t*-test was adopted to compare the differences of continuous variables between groups. For categorical variables, a χ test or Fisher’s exact test was applied at a two-sided significance level of 0.05. For the relative abundance of microorganisms, the Mann–Whitney U test was applied at a two-sided significance level of 0.05. The R package vegan was used for alpha- and beta-diversity analysis. A random forest model between the NP and the CP group was established by using the R package randomForest. The visualization of the above results and the plotting of the box plot were carried out by using the R package ggplot2. A multivariate binary logistic regression model was constructed using SPSS 26.0 with backward stepwise regression. The R package pROC was used to perform bootstrap repeated random sampling and the plotting of receiver operating curves (ROC).

## Data Availability

The raw sequence reads can be found under accession no. PRJNA1224408.

## SUPPLEMENTAL MATERIAL

Supplemental figure legends spectrum.01462-24-s0001.docx Legends for Figures S1 and S2.

Figure S1 spectrum.01462-24-s0002.tif Phylum-level beta diversity analysis.

Figure S2 spectrum.01462-24-s0003.tif Composition of the microbiota at different levels.

Table S1 spectrum.01462-24-s0004.xlsx Differential taxa between the NP and CP groups at different levels.

ASM does not own the copyrights to Supplemental Material that may be linked to, or accessed through, an article. The authors have granted ASM a non-exclusive, world-wide license to publish the Supplemental Material files. Please contact the corresponding author directly for reuse.
