# Supplementary material for: A nomogram prediction model for embryo implantation outcomes based on the cervical microbiota of the infertile patients during IVF-FET
Source: Microbiol Spectr. 2025 Mar 7;13(4):e01462-24. doi: 10.1128/spectrum.01462-24 (PMC11960138; doi:10.1128/spectrum.01462-24)
Supplement: Supplemental figure legends — Legends for Figures S1 and S2. [file spectrum.01462-24-s0001.docx]

**SUPPLEMENTAL FIGURE LEGENDS**

**Figure S1. Phylum-level beta diversity analysis.**

(A) Principal component analysis at the phylum level; (B) Principal coordinate analysis at the phylum level; NP, Non-pregnancy; CP, Clinical pregnancy.

**Figure S2. Composition of the microbiota at different levels.**

A, B, C, and D were the composition of the phylum, class, order, and family, respectively. When the number of taxa exceeds 20, only the top 20 taxa in relative abundance were labeled; NP, Non-pregnancy; CP, Clinical pregnancy.
