# Supplementary figures and images for: A nomogram prediction model for embryo implantation outcomes based on the cervical microbiota of the infertile patients during IVF-FET
Source: Microbiol Spectr. 2025 Mar 7;13(4):e01462-24. doi: 10.1128/spectrum.01462-24 (PMC11960138; doi:10.1128/spectrum.01462-24)

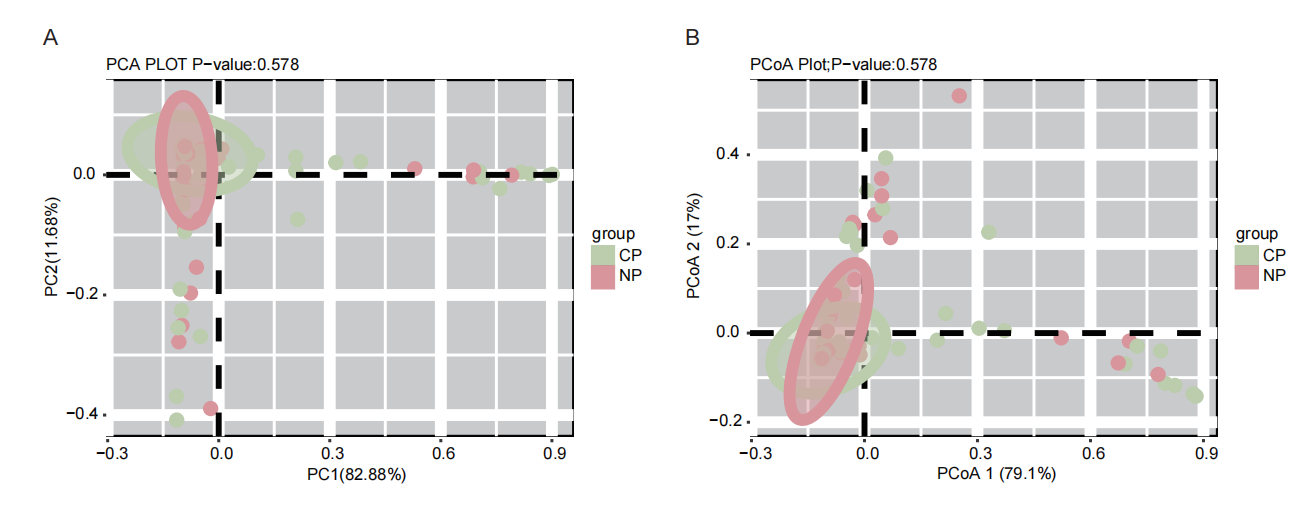

Supplement: Figure S1 — Phylum-level beta diversity analysis. [file spectrum.01462-24-s0002.tif]

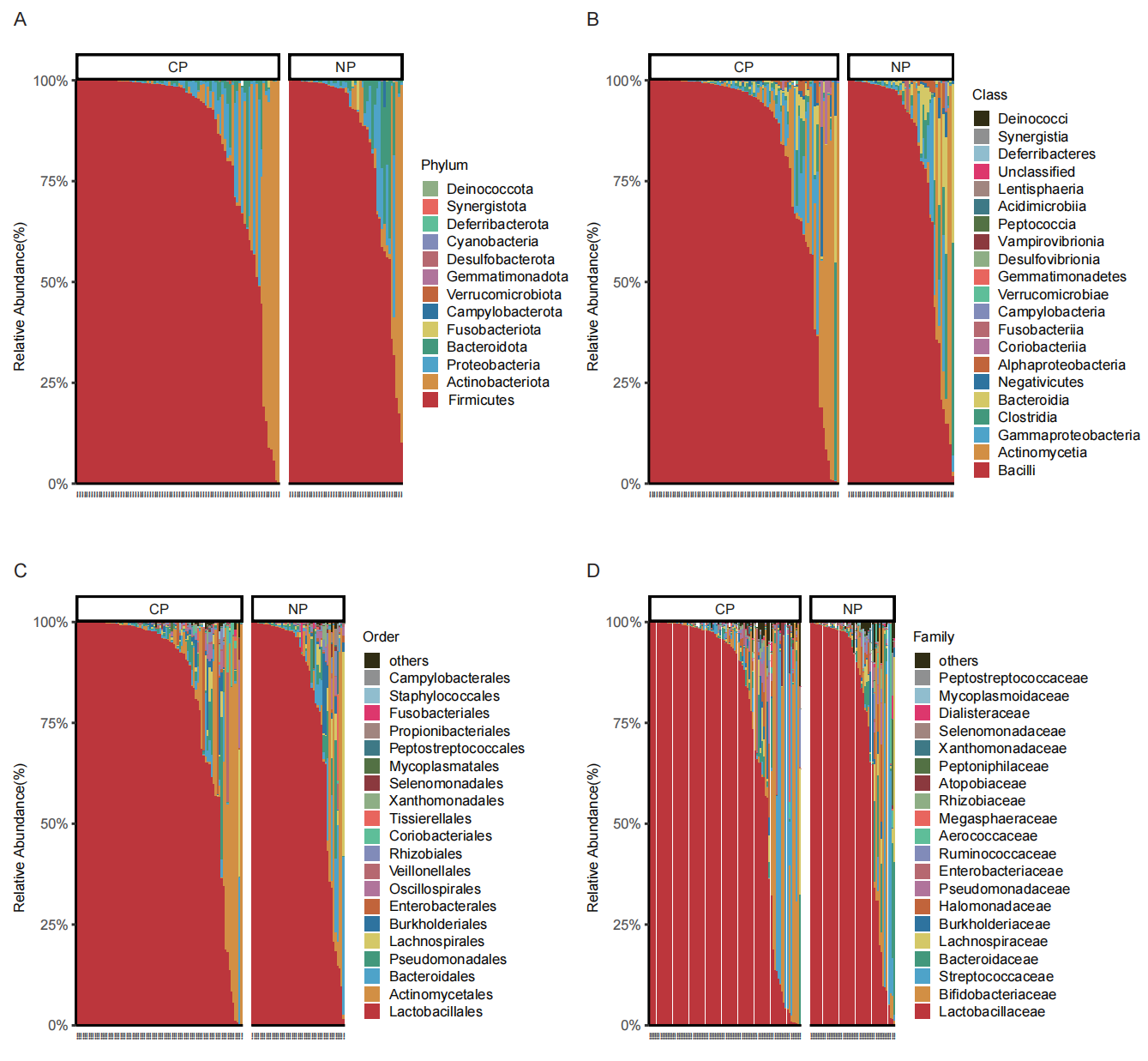

Supplement: Figure S2 — Composition of the microbiota at different levels. [file spectrum.01462-24-s0003.tif]
